# Tumescent Liposuction: A Review

**DOI:** 10.4103/0974-2077.44159

**Published:** 2008

**Authors:** Jayashree Venkataram

**Affiliations:** *Consultant Liposuction Surgeon, Venkat Charmalaya, Bangalore, Karnataka, India*

**Keywords:** Liposuction, tumescent anaesthesia, micro cannulae, local anaesthesia

## Abstract

Liposuction is a cosmetic procedure to remove fat. Liposuction may be performed either under general anaesthesia or under local anaesthesia. The procedure has been reported to be associated with significant morbidity and risk of mortality under general anaesthesia. Since the first description by Jeffrey Klein, dermatologic surgeons have made significant contributions in this field, and tumescent liposuction using microcannuale under local anaesthesia, is regarded as safe and effective. The author has performed over 200 liposuctions in the last four years in India and this article describes the procedure of microcannular tumescent liposuction in the light of her experience.

## INTRODUCTION

Liposuction is the surgical removal of subcutaneous fat by means of aspiration cannulae introduced through small skin incisions, assisted by suction. Synonyms used in literature include liposuction surgery, suction-assisted lipectomy, suction lipoplasty, fat suction, blunt suction lipectomy, and liposculpture.[1,2]

Liposuction is one of the most commonly performed cosmetic procedure today.[1] Dermatologists now perform about one third of these procedures in the United States and have pioneered many of the advances in liposuction, especially in the fields of ambulatory surgery and local anaesthesia.

In India, in the past decade since economic liberalization, the scenario has changed dramatically with respect to cosmetic procedures. Globalization, improved affluence, access to information via internet and television, and a high degree of awareness about health and beauty have all led to an increasing demand for aesthetic procedures. An increasing number of dermatologists are now performing aesthetic procedures. This article discusses the microcannular tumescent liposuction, which has emerged as the gold standard method in liposuction, in the background of our experience with about 200 patients over the last four years.

### Structure of fat

Subcutaneous fat is arranged in the form of lobules separated from each other by septae.[3–6] The fibrous septae consist of blood vessels, nerves, and lymphatics. Each lobule consists of fat cells, which consist mostly of triglycerides and fill up the cell almost entirely, pushing the nucleus to one side. It has been shown that during initial weight gain in any person, there is an increase in the size of the fat cell. With continued weight gain, there is, in addition, an increase in the fat cell number as mesenchymal stem cells get converted to fat cells. Diet and exercise have been shown to decrease the fat cell size, but not the fat cell number, which is referred to as “resistant fat”.

Liposuction is a method of reducing the fat cell number and thereby, the resistant fat. Liposuction removes the resistant fat by two mechanisms:

1. Removal of fat cells during suction [Figure 1]

**Figure 1:**
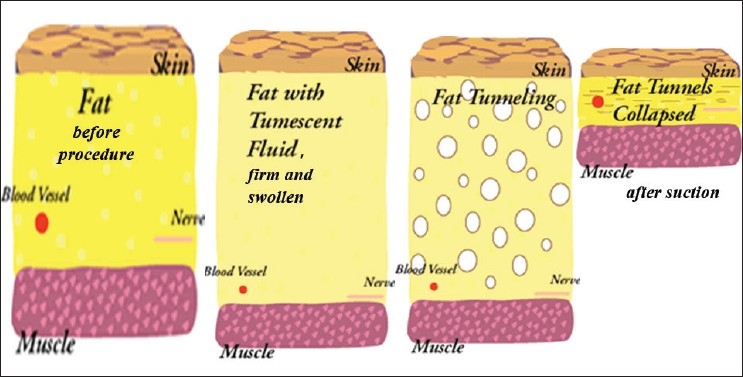
Principle of liposuction; fat before and after tumescence, and after aspiration
2. Damaging the fat cells by the to-and-fro motion of the cannula. These remaining damaged fat cells get absorbed slowly over 6–12 weeks and hence, the final result after liposuction is seen after 6–12 weeks, a fact which needs to be emphasised during counselling.

## HISTORY OF LIPOSUCTION

Liposuction was initially developed in the late seventies in Italy and France.[7,8] At that time, liposuction was performed under general anaesthesia without any introduction of fluid, hence, called “dry liposuction”. Later, a small amount of fluid was introduced into the fat (the “wet technique”). These methods were associated with much blood loss, and patients frequently required blood transfusions. In 1985, Dr. Jeffrey A. Klein, a dermatologist in California, revolutionised liposuction surgery when he developed the tumescent technique, which permits liposuction totally by local anaesthesia and with minimal surgical blood loss.[8] Further modifications such as power liposuction and ultrasonic liposuction have been introduced with variable results. Despite these advances, the tumescent technique remains the worldwide standard of care for liposuction.[1,2]

## METHODS OF LIPOSUCTION

### Microcannular tumescent liposuction

The word “tumescent” means swollen and firm.[8,9] This technique involves subcutaneous infiltration of large volumes of crystalloid fluid called Klein’s solution, which contains low concentrations of lignocaine and epinephrine, followed by suction-assisted aspiration of fat by using small aspiration cannulae called microcannuale [Figure 1]. The term *tumescent liposuction* specifically excludes the use of any additional anaesthesia, either intravenous or gaseous, and by definition, is a method for performing liposuction surgery with the patient totally under local anaesthesia.[1,2]

The procedure of microcannular tumescent liposuction consists of two steps:

- Induction of anaesthesia by tumescent anaesthesia:
  1. Making 4–8 small incisions called adits (1–3 mm in size)
  2. Introduction of a large amount (1–4 L) of Klein’s solution into the fat. Klein’s solution contains lignocaine, epinephrine, and large amounts of saline. The saline balloons the fat tissue, epinephrine causes vasoconstriction, thus, decreasing bleeding, and lignocaine induces local anaesthesia. This procedure usually lasts 45–60 minutes.
  3. Allowing the fluid to percolate uniformly through all layers, a process called detumescence, lasts 30 minutes.
- Aspiration of fat by microcannular liposuction:
  1. Sucking the fat out through microcannuale which are 1.5–3 mm in diameter. This is a slow process lasting 1–1½ hours.
  2. Leaving the incision wounds of cannulae open to drain out fluid. A small amount of fluid is left back in the tissue and is allowed to drain slowly over two days. This residual fluid provides analgesia in the immediate postoperative period.
  3. Applying compression bandages and sending the patient home without any hospital admission.

The procedure has the advantages of safety, lack of need for hospital admission, and rapid postoperative recovery time. However, the procedure is also slow, taking 3–4 hours to perform and also, the amount of fat that can be extracted is usually limited to about 4–5 litres.

Conventional liposuction using large cannulae under general anaesthesia, largely practised by plastic surgeons, is performed as follows:

- General anaesthesia is used.
- Introduction of a small amount of fluid into the fat.
- Making large incisions (1–1.5 cm) to introduce cannulae.
- Sucking out large amounts of fat, often 8–10 litres (called megaliposuctions), quickly in 1–2 h, through large cannulae (6 mm-1 cm in diameter).
- Suturing the incision wounds of cannulae.

The whole procedure lasts 2–3 hours. Thus, this method is quick, can remove large amounts of fat, and saves time for the surgeon. However, it has the following disadvantages:

1. As the method is under general anaesthesia, the patient has to be hospitalized, which adds significantly to the cost and the possibility of hospital-acquired infections.
2. General anaesthesia always has its risks.
3. The use of large cannulae causes greater damage to tissue and hence, increases the bleeding. This technique is associated with significant blood loss,[10,11] often needing blood transfusions.
4. There is a risk of side effects such as fat embolism, which can be potentially fatal.
5. Large cannulae need large incisions which have to be sutured and which heal with significant scars.
6. Recovery time is slow, as after any procedure under general anaesthesia.

Table 1 shows the comparison between the two methods.

**Table 1:** Comparison of techniques

	Conventional method	Tumescent method
Pain control	General anaesthesia	Local anaesthesia
Risks	Higher complication rate	Lower complication rate
Bleeding	Significant blood loss often requiring transfusion	Minimal blood loss
Postoperative period	Requires strong, parenteral pain-killers	Pain relief up to 24 h, needing only minimal, oral pain killers
Hospital admission	Needed	Outpatient procedure
Return to work	Days to weeks	1–3 days
Cannula size	Larger cannulae	Smaller cannulae
Sutures	Required	Not required
Speed	Quick	Slow
Amount of fat	Large, 8–10 litres	Smaller, 3–5 litres
Cost	Expensive because of hospital stay	Less expensive

### Other methods of liposuction

Power-assisted liposuction[12] with a reciprocating cannula is a new technology for liposuction and has some advantages. In powered liposuction, the reciprocating motion of the cannulae mimics the to-and-fro action of the surgeon’s cannula movement, decreasing the work of the procedure and is therefore, less tiring for the physician. In addition, it allows the surgeon to remove fat more completely in “tight” areas where forceful cannula movements are difficult because of physical space constraints (*e.g*., per umbilical and waist areas). While powered liposuction can help to remove fat quickly, it can do so only if large cannulae are used. Usually power-assisted liposuction also needs concomitant IM or IV narcotics and sedatives, as well as sometimes using nitrous oxide. These features therefore, negate the above mentioned advantages of tumescent liposuction (safety because it is done under local anaesthesia, and finesse because of the use of microcannuale).

### Ultrasound-assisted liposuction

Ultrasound-assisted liposuction (UAL) was introduced to damage the fat cells and thereby, facilitate the removal of fat.[13] However, the method had significant side effects such as burns of the skin. The damaged fat also lead to small cysts containing fluid called seromas. Ultrasound-assisted liposuction is associated with significant bruising and prolonged postoperative swelling. Most importantly, the ultrasound machines are expensive, increasing the cost of the procedure.

## INDICATIONS FOR LIPOSUCTION

Liposuction can be used in the following situations:[1,2]

1. Fat that is resistant to diet or exercise, located in any area of the body such as the abdomen, thighs, hips, neck, face, and under the chin.
2. Liposuction can be used for breast reduction. In men, gynaecomastia is an important indication for liposuction.
3. Liposuction has been found to be useful for noncosmetic indications also, such as hyperhidrosis of axillae[14] and lipomas.[15]

Table 2 shows our experience in Indian patients. It is interesting to note that there were more male patients for this procedure than women. Gynaecomastia is the most common indication in men, whereas the abdomen is the most common area in women. Gynaecomastia causes much social embarrassment in India, particularly at the time of weddings, when the bridegroom has to sit for religious ceremonies without wearing a shirt. Eighteen women also sought the procedure for their thighs, which reflects the increasing tendency of young women to wear tight jeans. As many as 18 patients came back for a second session, one patient for a third session, and one patient for a fourth session.

**Table 2:** Indications in 200 Indian patients (author's experience)

Indications	Men (n=113)	Women (n=87)	Total
Abdomen	45	37	82
Thighs	-	23	23
Chin	1	3	4
Arms	-	4	4
Buttocks	-	7	7
Flanks / Love handles	8	5	13
Breast	59	6	65
Axilla	-	2	2

## PRINCIPLE OF TUMESCENT ANAESTHESIA

The most important aspect of tumescent liposuction is that a local anaesthetic is used over a wide area to provide anaesthesia and analgesia, using a sufficient quantity of lignocaine far in excess of the conventional dosage. Conventional teaching has widely regarded, without adequate pharmacological proof, that the safe upper limit for lignocaine administration is 6 mg/kg body weight. In a radical departure from this conventionally accepted fact, Klein showed that in tumescent anaesthesia, much higher doses, even up to 45–55 mg/kg weight can safely be administered.[16–20] This is because in tumescent anaesthesia, the rate of absorption of lignocaine is slow, leading to smaller peak values and hence, lesser toxicity. The reasons for the slow absorption of lignocaine are:

- Subcutaneous fat has a low volume of blood flow.
- Lignocaine is lipophillic and is easily sequestered in fat.
- Diluted epinephrine in saline solution ensures vasoconstriction, thus, minimizing systemic absorption and bleeding.
- The large volume of tumescent solution itself compresses blood vessels by hydrostatic pressure.
- The very low dilution of lignocaine in Klein’s solution does not achieve the gradient required for systemic absorption.
- Most of the solution is removed during aspiration, minimizing the duration for absorption.

This slow absorption from subcutaneous fat has been likened to a slow release capsule, with the fat itself acting as the capsule!!

## PROCEDURE OF TUMESCENT LIPOSUCTION

### Patient selection

Proper patient selection is highly important—the ideal candidates are patients with localized deposits of fat, who are not grossly obese, without significant medical problems, and have realistic expectations.[1,2,5,17,21] Many patients seek consultation in the mistaken assumption that liposuction is a treatment for weight reduction. It should be clarified to them that liposuction is only for improvement of shape and any weight loss (which will be about 4–5 kg) is only incidental. There is no definite age or weight limit for patients to undergo liposuction.

The maximum amount of fat that can be removed safely by tumescent liposuction is probably about 4–5 litres.[10] Generally, it is advisable to avoid the so-called megaliposuctions as they are associated with complications.[1,2] The risk of side effects increases with removal of larger amounts of fat. Different areas such as the abdomen and the thigh or buttock are not generally combined in one session.[18] However, it is possible to treat both buttocks or both thighs in one session. If patient desires more than one area or needs more than 4–5 litres of fat removal, the procedure may be repeated any time after two weeks.

A thorough medical history with particular reference to history of bleeding diathesis, emboli, thrombophlebitis, infectious diseases, poor wound healing, and diabetes mellitus should be always taken. Patients with a medical history of these conditions need to be examined and cleared by a physician before undergoing liposuction. Liposuction is contraindicated in patients with severe cardiovascular disease, severe coagulation disorders including thrombophilia, and during pregnancy. The patient’s history should also include noting prior abdominal surgeries such as caesarean sections which produce scarring. A detailed drug history is essential. As lignocaine is metabolized by the liver, drugs that compete with it for metabolism by the cytochrome P_450_ enzyme system or displace lignocaine from plasma proteins can increase lignocaine blood levels and cause lignocaine toxicity.

The physician must perform a detailed physical examination to determine that the areas of planned for surgery are amenable to liposuction. In particular, any evidence of keloids, scars, or hernia should be looked into.

### Counseling

Counseling should include:[21]

- Discussion on different management options, including the role of diet and exercise.
- Detailed explanation about the surgical procedure, including possible postoperative complications.
- Specific instructions that full results would be seen after 6–12 weeks.
- Instruction that although the fat removed by liposuction does not normally come back, there may be recurrence of the problem if the patient puts on excessive weight. The importance of continued exercise and diet regulation should be stressed.
- Any allergies or medical condition that the patient may have should be recorded.
- As in any cosmetic procedure, the patient should not expect to achieve perfection.
- Patients should be told not to expect to lose any dramatic amount of weight loss with liposuction. Weight lost is equal only to the amount of fat removed, about 3–5 kg.
- Patients should also understand that liposuction does not improve cellulite or the striae. Patients can also be assured that there is no likelihood of loose skin hanging in the operative area due to the elasticity of skin. Abdominoplasty is usually not necessary for abdominal contouring but is only necessary if a large amount of excess skin or muscle laxity is present.[22,23] The recent introduction of skin tightening machines has also helped in the management of any mild laxity.

### Preoperative instructions

These are routine and include:[21]

- Routine blood investigations such as blood counts, bleeding and clotting time, prothrombin time, blood sugar, liver function tests, HbS Ag, HIV-ELISA, and ECG.
- Advice to stop smoking and oral NSAIDs as smoking increases intraoperative bleeding.
- Preoperative tranquillizers such as diazepam or lorazepam on the night before surgery to relieve any anxiety.
- Injection Vitamin K to minimize postoperative bruising.

**Figure 2:**
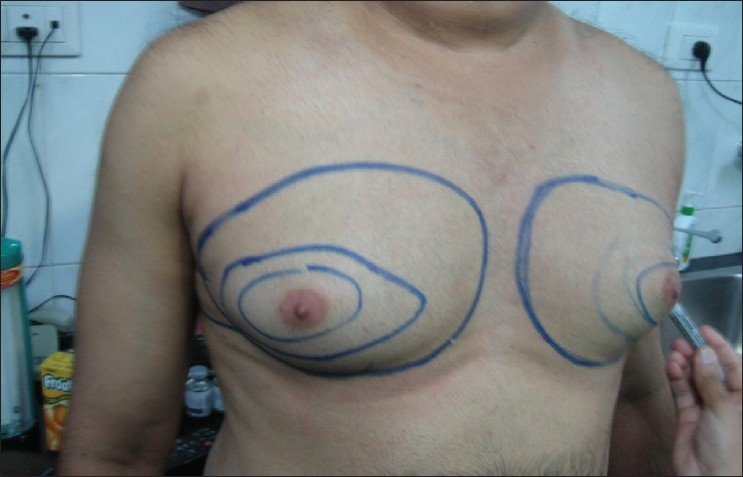
Topographic marking

On arrival on the day of the surgery, patients are administered preoperative antibiotics such as cephalexin, and a tranquillizer such as oral lorazepam 1 mg. Oral Clonidine 0.1 mg is also administered to prevent epinephrine-induced tachycardia and as an adjuvant anxiolytic drug. The area for liposuction is topographically marked with marker ink to delineate the bulges and asymmetry [Figure 2]. Preoperative photography is vital.

### Monitoring

Baseline vital signs including blood pressure and heart rate, are to be recorded preoperatively and monitored intraoperatively. Pulse oximeter monitoring is essential. Medical personnel trained in resuscitation, preferably an anaesthetist, should be available on the premises.

### Tumescent anaesthesia

This is a very important and vital step. Proper tumescence will ensure painless and smooth aspiration.

**Figure 3:**
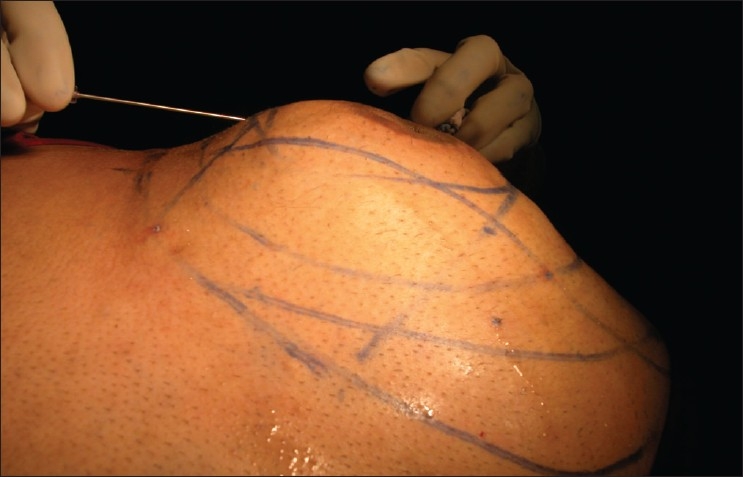
Infiltration of tumescent fluid
- Detumescence: It is important to wait for about 30 min after tumescence for the infiltration fluid to percolate properly and its full pharmacological effects to take effect. This is indicated by a slight decrease in firmness and the ability to grasp the skin.

- Adits are small holes made for insertion of infiltration cannulae. These are done with 1.5–2 mm dermal punches in different locations of the area under infiltration anaesthesia with 1 mL of 2% lignocaine. The number of adits needed depends on the area involved. About 6–8 adits are normally needed for the abdomen.
- Tumescent fluid is prepared as follows: The usual tumescent solution concentration used is 0.05–0.1% lignocaine and the concentration of epinephrine is at 1:1,000,000–1.5:1,000,000. As the lignocaine solution is acidic, 10 meq of sodium bicarbonate solution is added to one litre of tumescent solution to raise its pH and to prevent stinging. The acceptable maximum dose of lignocaine is 55 mg/kg for most patients, although we have used dosages up to 57 mg/kg in our patients. If higher concentrations are needed, small amounts of fluid can be reintroduced after partial aspiration to avoid excessive dosing. The recommended concentration of epinephrine in tumescent solutions is 0.25–1.5 mg/L. The total dosage of epinephrine should not exceed 50 µg/kg.
- Infiltration of tumescent fluid: The delivery system for tumescent solution consists of infusion bags, infiltration pressure cuffs, an infiltration pump to hasten delivery of the fluid, and infiltration cannulae 0.5–1 mm in size. About 2–3 litres of fluid are infiltrated gradually in different directions, first into deeper layers of fat and then, into the superficial layers. The end point is a firm feel of the skin which makes the skin swollen, and difficult to grasp [Figure 3]. It is important to be slow and to avoid jerky, sudden movements to avoid pain. Normally it takes about 1 to 2 h for proper anaesthesia.

## ASPIRATION

**Figure 4:**
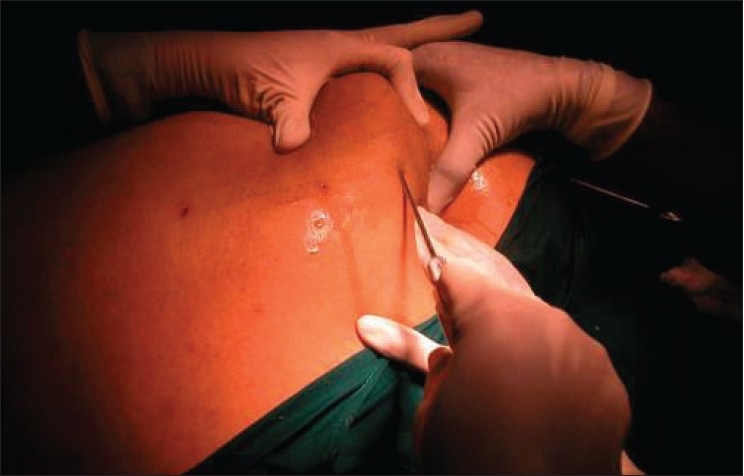
Aspiration of fat

**Figure 5:**
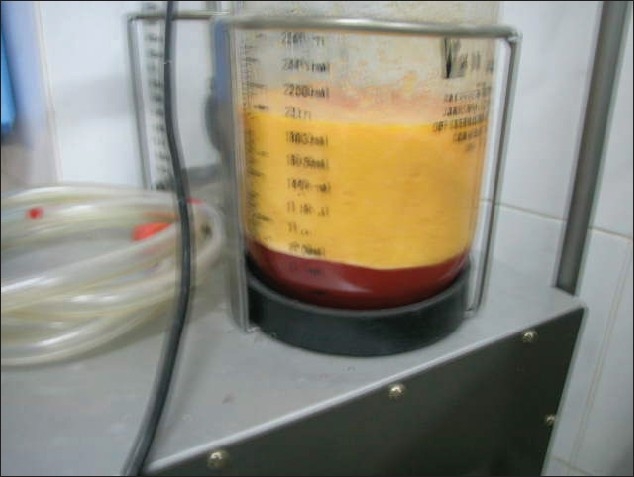
Fat aspirated; note the small amount of blood

The most important aspect of proper aspiration is the slow, repeated, to-and-fro movement of the cannulae.[24,25] The cannulae are of different sizes, varying in diameter from 1 to 2.5 mm. Cannulae larger than 4.5 mm in diameter are not used as they cause more tissue damage and are associated with the risk of embolism and bleeding. A smaller cannula (1 mm) is first used to create tunnels in the fat. Cannulae of gradually increasing diameter are then employed to aspirate fat. Deeper layers of fat are aspirated first and then the superficial layers. The direction of the handles is always parallel to the skin and is never vertical [Figure 4]. The nonoperating hand is used as a guide to push the fat in the direction of aspiration and also, to feel the tip of the cannula to prevent damage to the overlying skin or underlying structures. It is also important to avoid skin trauma at the adit to ensure proper healing of the adits. Care should be exercised to ensure uniform aspiration in all areas and to avoid excessive aspiration from a given area to avoid dimpling and asymmetry. Different areas are aspirated and then compared for symmetry and regularity. One great advantage of tumescent anaesthesia is that because the patient is conscious, (s) he will feel the pain and warn the surgeon if the cannula is moved deep into the muscle or into the surrounding unanaesthetized area. Also, the patient is in a position to sit or stand so that the surgeon can compare the two sides for symmetry. The process of aspiration normally takes between 90 minutes to two hours and about 3–5 litres of fat are aspirated. Blood loss is minimal and does not exceed 30–50 mL if the tumescence is proper [Figure 5]. It is important to keep the patient engaged by having a television or music in the theatre during the entire procedure.

Average amounts aspirated from different areas in our patients are shown in Table 3. The maximum amount of fat aspirated in our experience is 4.2 litres.

**Table 3:** Average fluid aspiration in different areas in Indian patients

Sl No	Male	Female	Average (mL)
1. Abdomen	45	-	3832
		37	3611
2. Breast	59	-	2189
		06	4200
3. Thighs		23	4157
4. Arms		04	2975
5. Flanks	08	-	3038
		05	2350
6. Buttocks		07	2965
7. Chin	01	03	450
			237
8. Axilla		02	1100

### Postoperative dressing and follow-up

**Figure 6:**
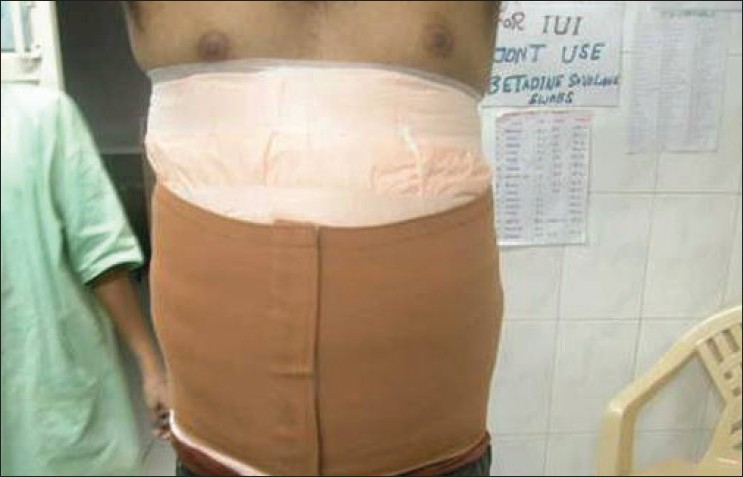
Postoperative pressure dressing

Postoperative dressing is a very important step in tumescent liposuction. An important feature of the tumescent procedure is that some amount of the fluid is still left behind at the end of the procedure, which ensures anaesthesia in the immediate postoperative period, minimizing the need for potent oral analgesics. This fluid drains out in 3–5 days to facilitate which the adits are not sutured and are allowed to heal by secondary intention. Tight pressure bandages are essential to ensure proper drainage of the tumescent fluid [Figure 6]. Two layers of pressure dressing (called bimodal compression) are put in place to ensure tight compression in the first two days.[26,27] Dressings are removed on the first postoperative day and the adits are opened again, if necessary, to ensure proper drainage. Improper drainage increases the possibility of panniculitis, secondary infection, and irregularity. Postoperative analgesics and antibiotics are continued. The pressure in the dressing is decreased after three days and continued for a minimum of two weeks. The patient is advised to come for follow-up for daily dressing for three days. It is important to note that while the patient can return to normal sedentary work in 1–2 days, exercise and undue exertion should be avoided for at least ten days

## RESULTS

**Figure 7:**
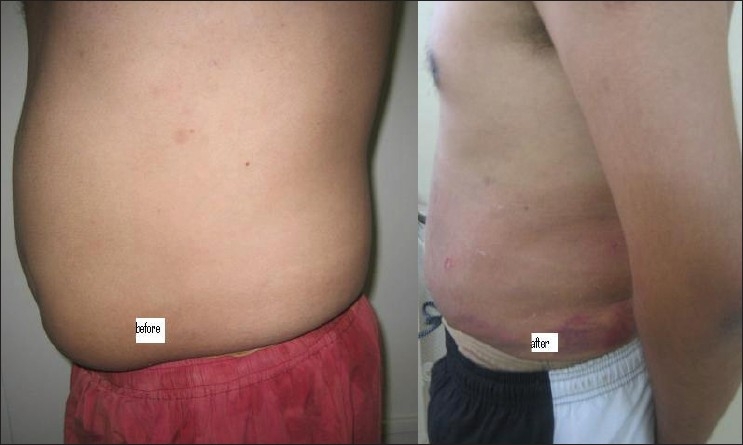
Liposuctions results, case 1

**Figure 8:**
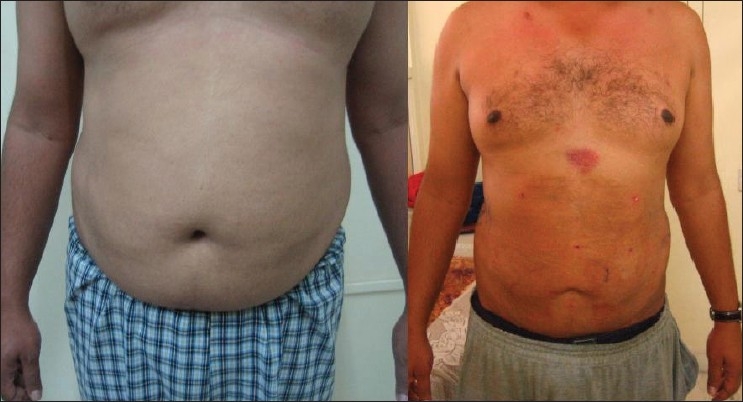
Results of case 2

**Figure 9:**
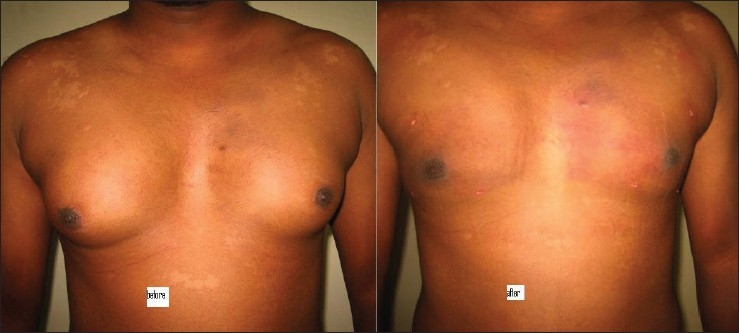
Liposuctions results, case 1

Figures 7–9 show the results in the regions of the abdomen and the chest in three patients. It should be noted that there is some improvement seen immediately after surgery. However, more improvement is seen over the next three days as residual fluid drains out and also, with continued absorption of damaged fat over 4–6 weeks. Hence, final results are seen at the end of six weeks.

## COMPLICATIONS

Tumescent anaesthesia is a remarkably safe procedure if all the essential steps are adhered to.[17,28–30] In the author’s experience of nearly 200 cases, side effects have been rare.

- Postoperative pain: This is minimal in the first two days because of the persistent anaesthetic fluid in the tissue. Mild oral analgesics such as paracetamol are all that are required. Mild tenderness at the site of adits may be felt over 3–5 days. An antibiotic cream (such as fucidic acid or mupirocin) may be prescribed for application at the sites of the adits.
- Postoperative oedema over dependent parts (such as legs and genitals) may occur and is due to the inflammation caused by the aspiration movements. It is minimized by using small cannulae and proper postoperative dressings.
- Postoperative syncope is common and is vasovagal in origin due to the sudden release of pressure while removing the tight bandages. It is easily avoided by releasing the bandage in the supine position and asking the patient to get up gradually. Mild tenderness is also expected over the adit sites.
- Postoperative ecchymoses may occur which usually disappears spontaneously over a week. This is common in hypertensive patients and hence, proper blood pressure control is essential.
- Diffuse tenderness and induration can occur if the drainage is improper.
- Panniculitis and fat necrosis are rare, but they may occur in diabetics. Hence, it is important to ensure proper diabetic control.
- Postoperative infection is rare if proper aseptic precautions are followed.
- Seroma formation: Seromas are cystic swellings which occur due to aggressive superficial fat aspiration. They are more common in ultrasound-assisted aspiration but were seen in only two patients encountered by the author.
- Irregularity and asymmetry can occur if the amounts of fat aspirated are different in different areas and if pressure garments are not worn properly. This is common over the chest and upper abdomen. It is also common in men treated for gynaecomastia and in patients in whom large cannulae have been used to remove fat quickly.
- Pigmentation is common in Indian patients over the adit scars, although none of our patients experienced any keloids.

## SAFETY OF TUMESCENT LIPOSUCTION

Several serious complications have been reported with conventional liposuction done under general anaesthesia. These include pulmonary embolism, excessive blood loss, hemorrhagic necrosis of fat, and even, death.[28,29] These complications have been reported mostly in patients in whom liposuction is combined with other procedures such as abdominoplasty, or more than one area have been treated, and in megaliposuctions.

However, these complications are extremely rare in tumescent liposuction and the safety of tumescent liposuction has been well documented in literature. In our experience of 200 cases, no patient had any serious side effect and all patients recovered without any untoward incident. Extensive reviews have been carried out to establish the safety of the procedure and different parameters such as the amount of fat aspirated, type of anaesthesia, facility for surgery, and speciality of operating surgeon have all been studied in large reviews. It is important to note that while mortality has been reported with conventional liposuction, not a single death has been recorded after tumescent liposuction.[1,2,17,30–32] These studies are discussed in detail below.

In a survey of 9478 liposuction cases[31] performed by dermatologic surgeons, the risk of systemic complication was found to be as low as 0.07%. Five patients had “excessive” intra- or postoperative blood loss, and two patients had infection. There were no reported cases of disseminated intravascular coagulation, fat emboli, perforated viscus, thrombophlebitis, or death. The risk of local complications was also small. Of these, the most common were postoperative contour irregularities (2.1%), hematoma (0.47%), and persistent postoperative oedema (46%). A later (1995), more extensive survey of data on 15,336 patients undergoing tumescent liposuction also did not find any serious complications.[32]

In 1999, a study by Coleman determined whether the specialty of the physician had an effect on the incidence of malpractice claims. The study showed that < 1% of the defendants were dermatologic surgeons, even though dermatologic surgeons performed about 33% of liposuctions in the US. In 2002, in a national survey of over 66,000 liposuction cases performed using the tumescent anesthesia technique, no deaths were reported and the rate of serious adverse events was 0.68 per 1000 cases.[33] A review of the State of Florida adverse event data revealed that there were no tumescent anesthesia-related liposuction deaths.[34] In contrast, there were two deaths related to liposuction under general anaesthesia. Safety of office-based liposuction as opposed to hospital-based liposuction too has been well documented. It was found that hospital-based liposuction had three times the rate of malpractice settlements when compared with office-based liposuction surgery.[35–37]

Thus, these data have conclusively established the safety of this procedure, particularly when performed by dermatologic surgeons and as an office-based surgery. Hence, tumescent liposuction is now regarded as the gold standard method for liposuction.

## SUMMARY

Tumescent liposuction is a safe and effective procedure when performed in trained hands in a proper setting. Experience and training of surgeon, proper selection of cases, and proper technique in anaesthesia and aspiration are all important to get optimal results. It is important to keep in mind that, as in any cosmetic procedure including liposuction, a final safe and satisfactory result is far more important than quick results.

## MULTIPLE CHOICE QUESTIONS

1. Liposuction is performed to remove fat for:
  - aesthetic purpose
  - weight reduction
  - to treat diabetes
  - all the above
2. In tumescent technique, Klein's fluid contains the following:
  - pethidine
  - lignocaine and saline
  - lignocaine, epinephrine, and saline
  - lignocaine, epinephrine, saline, steroid
3. The safe upper limit for removing fat in tumescent liposuction is:
  - 1 litre
  - 5 litres
  - 10 litres
  - any amount
4. The most important feature of tumescent liposuction as opposed to conventional liposuction is:
  - fluid is introduced into fat
  - is performed under local anaesthesia
  - bimodal compression is used
  - all the above
5. The safe upper limit for dosage in tumescent liposuction is:
  - 6 mg/kg
  - 12 mg/kg
  - 45-55 mg/kg
  - None of the above
6. Detumescence is necessary to:
  - allow percolation of fluid through out all layers of fat
  - allow the patient to relax
  - allow the surgeon to relax
  - facilitate metabolism of lignocaine and thus, prevent its toxicity

## ANSWERS

1:a, 2:c, 3:b, 4:d, 5:c, 6:a
